# Long-term QT prolongation in monkeys after doxorubicin administration at doses similar to breast cancer therapy

**DOI:** 10.3389/fcvm.2023.1247273

**Published:** 2023-12-12

**Authors:** George M. Bodziock, Giselle C. Meléndez

**Affiliations:** ^1^ Department of Internal Medicine, Section of Cardiovascular Medicine, Cardiac Electrophysiology, Wake Forest University School of Medicine, Winston-Salem, NC, United States; ^2^ Department of Pathology, Section on Comparative Medicine, Wake Forest University School of Medicine, Winston-Salem, NC, United States

**Keywords:** antracyclines, QT prolongation, monkeys, repolarization reserve, cardiomyopathy, Torsade de point

## Abstract

**Background:**

Studies in small animals and human patients have suggested that anthracyclines may prolong cardiac repolarization, or at least inhibit repolarization reserve, predisposing to QT prolongation and dangerous arrhythmias such as Torsades de pointes. This association in humans is difficult to confirm due to multiple confounding variables such as the presence of other medications and concurrent illness.

**Objectives:**

Identify a long-term association between anthracycline administration and repolarization prolongation in nonhuman primates, which can be measured as prolonged QT/QTc intervals on surface electrocardiogram.

**Methods:**

Five female African Green monkeys (AGMs) aged 13 ± 1 years received Doxorubicin (Dox) at doses similar to women treated for breast cancer (30–60 mg/m^2^/biweekly IV, total cumulative dose: 240 mg/m^2^) and underwent 12-lead electrocardiogram (ECG) before and 15 weeks after the final dose of Dox treatment. A blinded paired analysis was performed on ECG derived heart rate (HR), QRS, QT and QT corrected for HR (QTc) interval durations.

**Results:**

After Dox, all monkeys exhibited increased QT (BL: 323.2 ± 27.4 ms vs. Post-Dox: 366.4 ± 18.7 ms, *p* = 0.002) and QTc (BL: 440.2 ± 22.8 ms vs. Post-Dox: 500.8 ± 22.0 ms, *p* = 0.009) intervals, without any significant changes in HR or QRS duration (*p* = 0.92 and *p* = 0.47 respectively).

**Conclusions:**

AGMs treated with Dox exhibited long-term QT and QTc prolongation, along with the expected cardiotoxicity (LVEF decrease). While similar findings were shown in small animal studies, confounders make human association difficult to prove. Our finding provides a valuable intermediary step, showing direct effect of Dox on repolarization in nonhuman primates.

## Introduction

Certain antineoplastic therapies can increase the risk of cardiac arrhythmias by causing cardiac repolarization abnormalities, which can be measured as electrocardiogram (ECG) derived QT and corrected QT (QTc) prolongation (1). While anthracyclines have not been classically associated with QT prolongation, a recent study of adult survivors of childhood cancer, who were treated with anthracycline, demonstrates that these patients exhibit QT prolongation with an inverse association with left ventricular (LV) dysfunction (1, 2). In addition, studies in both animals and human patients have suggested anthracyclines may prolong cardiac repolarization, or at least inhibit repolarization reserve, predisposing to QT prolongation and dangerous arrhythmias such as Torsades de pointes (TdP) (1, 3). Whether anthracyclines are the culprit of these side effects is difficult to prove in humans due to confounding factors such as comorbid conditions, electrolyte disturbances, and use of other QT prolonging drugs (1). A clearer association was demonstrated in a small animal model showing that Doxorubicin (Dox, a frequently used anthracycline) predisposed to QT interval prolongation by reducing cardiomyocyte repolarization reserve (3).

We have previously reported that African Green monkeys exhibit cardiac magnetic resonance (CMR) derived myocardial fibrosis and LV dysfunction after receiving Dox at doses similar to those experienced by women treated for breast cancer (4). For this same group of monkeys, electrocardiograms were collected (ECG) at baseline and long-term (15 weeks) post-dox administration, giving us the unique opportunity to assess QT/QTc intervals in nonhuman primates, without comorbid conditions or other factors that may induce ECG abnormalities, long-term after Dox monotherapy. In addition, we looked for an association with myocardial fibrosis, a cardinal underlying characteristic of LV dysfunction. This is important because post-chemotherapy patients may benefit from QT monitoring long-term after treatment.

## Methods

### Animals

This study conformed to the principles of the National Institutes of Health and all protocols were approved by Wake Forest University (WFU) Animal Care and Use Committee. Five female premenopausal AGMs (*Chlorocebus aethiops sabeus*), part of a multigenerational pedigreed colony), aged 13 ± 1.3 years—equivalent to a 39-year-old woman- were used in this study; the lifespan of a AGM in the WFU colony is −26 years representing 1/3 of the lifespan of women in the US (−78.7 years) (5). Animals were housed in climate-controlled indoor pens, with 12-h light/dark cycles and food and water *ad libitum*. Animals were fed a commercial laboratory primate chow (Laboratory Diet 5,038, St. Louis, MO) with daily enrichments of fresh fruits and vegetables.

### Study design

**Figure 1:**
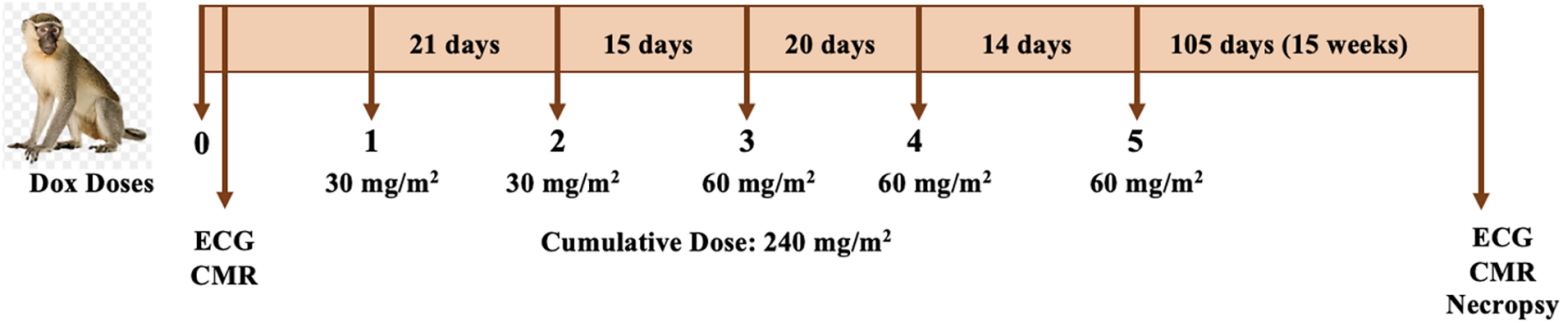
Study design. Animals underwent ECG and CMR imaging before and 25 weeks after of initiation of chemotherapy. Dox doses ranged between 30 and 60 mg/m^2^ to a total cumulative dose of 240 mg/m^2^. The post-treatment ECG and CMR was conducted one week prior to necropsy.

Animals underwent ECG measurement and cardiac magnetic resonance imaging (CMR) before and 15 weeks after the last dose of Dox treatment (Figure 1), which consisted of two initial doses of 30 mg/m^2^ and three doses of 60 mg/m^2^ given via vascular access port (VAP) every 17 ± 3.5 days (total cumulative dose: 240 mg/m^2^). At experimental endpoint, 25 weeks after initial ECG, euthanasia was induced in accordance with the American Veterinary Medical Association (AVMA) guidelines. While under anesthesia, a catheter was placed in a peripheral vein and euthanasia solution was administered at a dose of sodium pentobarbital −100 mg/kg IV. Electrolytes levels (calcium, phosphorus, sodium, potassium, and chloride) were acquired at baseline and post-Dox treatment.

Electrocardiograms were recorded using a GE Mac 5,500 Resting ECG system, generating a standard 12 lead ECG. Heart rates and intervals were measured and analyzed automatically by the GE system. By default, this system used the historic Bazett formula to correct the QT interval for heart rate, generating the QTc interval. Importantly, the Bazett formula correction was used in this analysis because it has demonstrated to be superior for QT interval correction to detect myocardial disease in nonhuman primates over the Fridericia and Framingham formulas (6).

### Statistical analysis

Descriptive statistics were calculated for all variables in Dox-treated AGMs at baseline and 15 weeks post-Dox treatment and compared to baseline values. ECG and electrolyte parameters were compared paired by t-tests and Pearson correlation was used to assess the association between QTc and extracellular volume (ECV), a CMR marker of myocardial fibrosis and left ventricular ejection fraction (LVEF). All analyses were performed using Graph Pad Prism version 7 for Windows (San Diego, CA).

## Results

**Figure 2:**
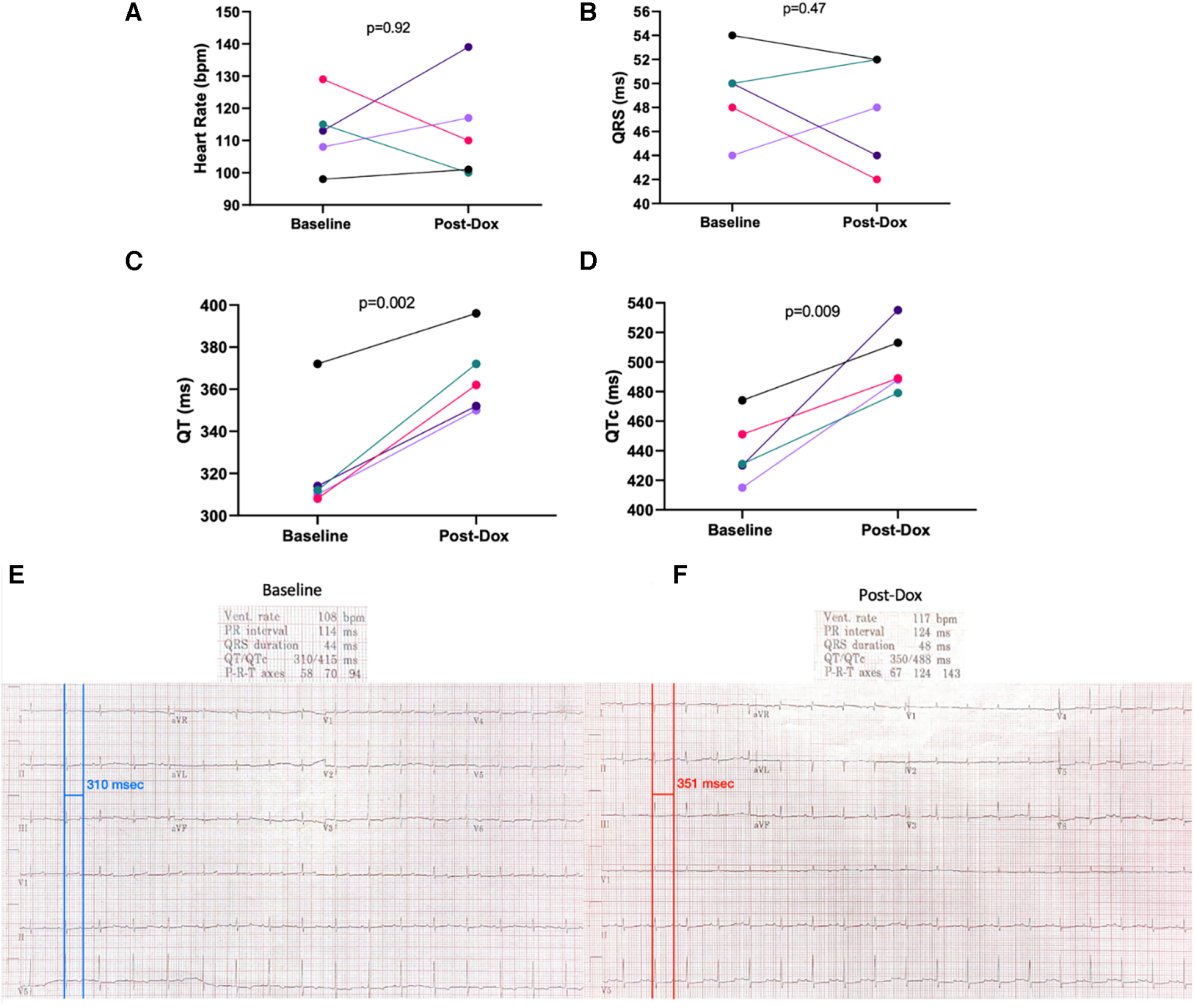
ECG rate and intervals at baseline and post-Dox treatment. (**A**) Heart rate in beats per minute (**B**) QRS duration in milliseconds (**C**) QT duration in milliseconds (**D**) QTc duration corrected for heart rate in milliseconds. Representative (**E**) Baseline and (**F**) Post-Dox ECG Traces. Red and blue lines denote manually measured QT intervals, which correlate with automated measurements. The tracings are shown with the machine derived rates, intervals, and axis above. QT interval prolongation most easily noted on the lead II rhythm strip.

**Figure 3:**
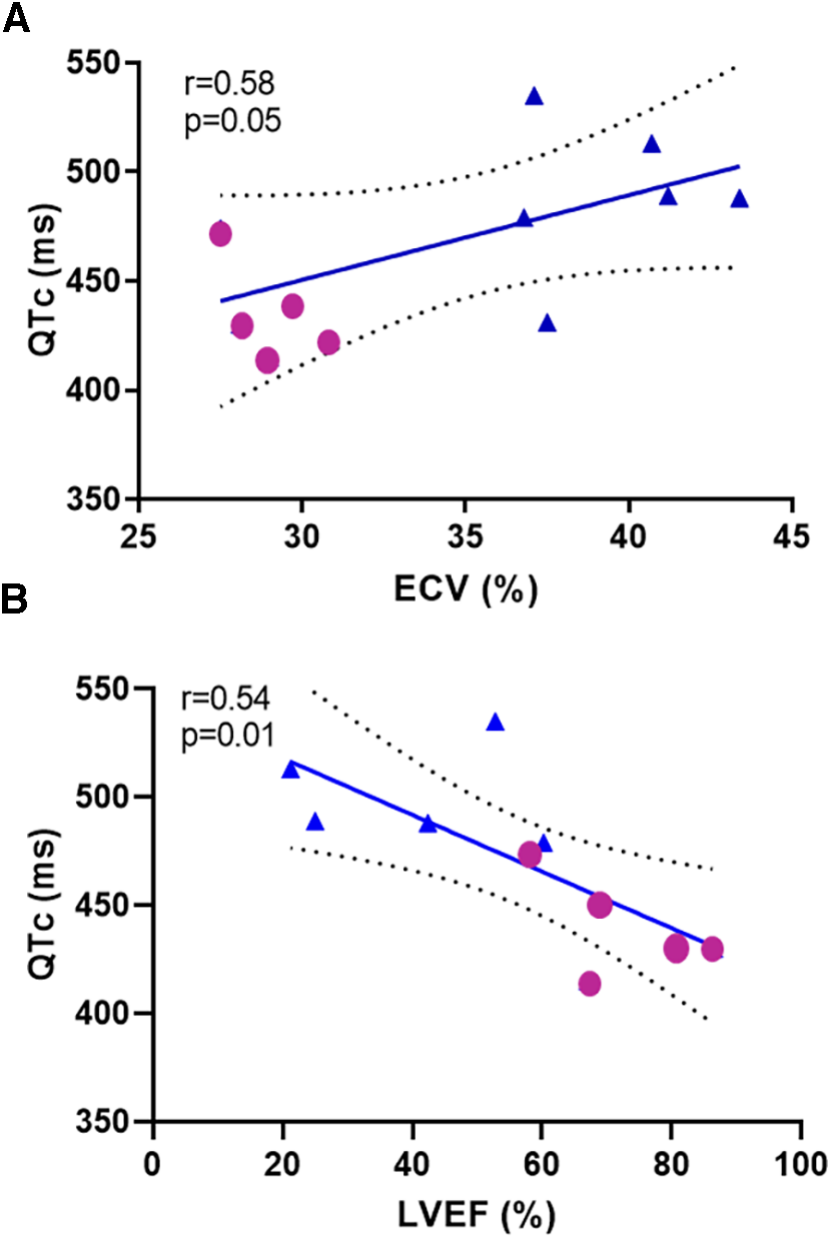
Correlation analysis between repolarization and (**A**) extracellular volume ECV and (**B**) left ventricular ejection fraction (LVEF) at baseline and post-Dox treatment. Repolarization demonstrated by QTc interval measured in milliseconds, ECV and LVEF.

After 15 weeks, post-Dox AGMs exhibited no significant difference in average resting heart rate (baseline: 112 ± 11 bpm vs. post-Dox: 113 ± 16 bpm; *p* = 0.46), PR interval (baseline: 120 ± 18 ms vs. post-Dox 116 ± 16 ms; *p* = 0.30), or QRS interval (baseline: 49 ± 3.6 ms vs. post-Dox: 47 ± 4.6 ms; *p* = 0.23). However, there was a statistically significant increase in QT (baseline: 323 ± 27 ms vs. post-Dox: 366 ± 19 ms; *p* = 0.001) and QTc (baseline: 440 ± 23 ms vs. post-Dox: 500 ± 23 ms; *p* = 0.004) intervals. These increases in QT and QTc intervals were observed in every animal, despite no significant change in other ECG parameters (Figure 2). An example of a baseline and post-Dox ECG tracings for one of the animals is also shown in Figure 2. Notably, we also found a moderate positive correlation between QTc and ECV (*r* = 0.58; *p* = 0.05) and a moderate inverse correlation between QTc and LVEF (*r* = 0.56; *p* = 0.01) (Figure 3). Electrolyte panels at baseline and post-Dox treatment demonstrate a significant increase in phosphorus and sodium blood levels (*p* = 0.0001 and 0.02, respectively, (Table 1). However, animals were asymptomatic (no diarrhea, vomiting, hyporexia, or signs of dehydration). As described previously, there was a significant decrease in body weight (BW) and body surface area (BSA) in these animals (4). None of the animals experienced sudden cardiac death, witnessed syncope, or other clear evidence of significant cardiac arrhythmias.

**Table 1:** Electrolyte values before and post-Dox treatment.

	Baseline (Mean ± STDEV) *n* = 5	Post-Dox (Mean ± STDEV) *n* = 5	*p*-values
Calcium (mg/dl)	8.58 ± 0.32	8.74 ± 0.42	0.17
Phosphorus (mg/dl)	3.62 ± 0.47	5.28 ± 0.61	0.0001
Sodium (mEq/L)	144.4 ± 2.40	146.8 ± 1.78	0.02
Potassium (mEq/L)	3.84 ± 0.16	4.06 ± 0.52	0.41
Chloride (mEq/L)	107.2 ± 2.28	106.2 ± 1.78	0.58

Dox, doxorubicin; STDEV, standard deviation.

## Discussion

We used Dox as monotherapy at doses and intervals similar to adjuvant chemotherapy in an AGM model that provided a valuable intermediate step between small animals and human patients. Our results suggest that Dox induces long-term repolarization prolongation—15 weeks after the last dose of Dox which roughly represents 55–60 weeks in a human—despite no significant differences in heart rate, and in the absence of confounding comorbidities such as malignancy, other medications, or electrolyte imbalances. These results suggest a direct effect of Dox on cardiomyocyte repolarization in AGMs, as was convincingly demonstrated by Milberg et al. in rabbits (3). We also found correlation between ECV and QTc (Figure 3A); for which similar associations have been shown previously (7), demonstrating the potential contribution of diffuse cardiac fibrosis to QT interval prolongation. Whether QT prolongation may precede ECV increase may be explored in future studies. Similarly, there was an expected inverse correlation between LVEF and QTc (Figure 3B). Perhaps early measures of repolarization may even forecast the development of cardiotoxicity and myocardial fibrosis after Dox administration. Importantly, QTc prolongation occurred independently of hypocalcemia or hypokalemia.

Our results should be interpreted in the context of the following limitations: first, our animals started this trial with a relatively healthy myocardium and no malignancy, in contrast to the typical co-morbidities exhibited by post-menopausal women and breast cancer patients. While this is a potential caveat to interpreting the results of this study, it should be noted that many patients who initiate breast cancer therapy are pre-menopausal and likely do not exhibit such co-morbidities, and are therefore representative, in part, of the AGM model characteristics for this study (8). Secondly, there were no observed clinical consequences related to the prolonged myocardial repolarization. Sodium and potassium levels did not change with Dox treatment. While magnesium levels were not measured, the animals in the study were under closely controlled settings without interference from any underlying medical illnesses or other medications, and there were no observed episodes of diarrhea or gastrointestinal distress to suggest electrolyte abnormalities.

In conclusion, even though anthracyclines have only been weakly associated with QT prolongation in humans, mostly through case reports (1), our findings demonstrate that Dox administration is associated with long-term abnormal repolarization, measured as QT prolongation, after the end of chemotherapy treatment in nonhuman primates. This supports close monitoring of cardiac repolarization in patients long-term after treatment with anthracyclines, especially with coadministration of QT prolonging agents such as antibiotics and antiemetics, since reduced repolarization reserve may not manifest with significant QT prolongation until other common proarrhythmic drugs are present.

## Data Availability

The original contributions presented in the study are included in the article/Supplementary Material, further inquiries can be directed to the corresponding author.
